# Estrogen Regulated Genes Compel Apoptosis in Breast Cancer Cells, Whilst Stimulate Antitumor Activity in Peritumoral Immune Cells in a Janus-Faced Manner

**DOI:** 10.3390/curroncol31090362

**Published:** 2024-08-24

**Authors:** Zsuzsanna Suba

**Affiliations:** Department of Molecular Pathology, National Institute of Oncology, Ráth György Str. 7-9, H-1122 Budapest, Hungary; subazdr@gmail.com; Tel.: +36-1-224-86-00; Fax.: +36-1-224-86-20

**Keywords:** antiestrogen, breast cancer, cancer therapy, DNA damage, DNA repair, endocrine disruptor, estrogen receptor, growth factor receptor, immune reaction, mutation

## Abstract

**Background**: Breast cancer incidence and mortality exhibit a rising trend globally among both premenopausal and postmenopausal women, suggesting that there are serious errors in our preventive and therapeutic measures. **Purpose:** Providing a series of valuable, but misunderstood inventions highlighting the role of increasing estrogen signaling in prevention and therapy of breast cancer instead of its inhibition. **Results:** 1. Breast cells and breast cancer cells with germline BRCA1/2 mutations similarly show defects in liganded estrogen receptor (ER) signaling, demonstrating its role in genomic instability and cancer initiation. 2. In breast tumors, the increased expression of special receptor family maybe an effort for self-directed improvement of genomic defects, while the weakness or loss of receptors indicates a defect requiring medical repair. 3. ER overexpression in breast cancer cells is capable of strengthening estrogen signaling and DNA repair, while in ER negative tumors, HER2 overexpression tries to upregulate unliganded ER activation and genome stabilization. 4. ER-positive breast cancers responsive to endocrine therapy may show a compensatory ER overexpression resulting in a transient tumor response. Breast cancers non-responsive to antiestrogen treatment exhibit HER2-overexpression for compensating the complete inhibition of hormonal ER activation. 5. In breast tumors, somatic mutations serve upregulation of ER activation via liganded or unliganded pathway helping genome stabilization and apoptotic death. 6. The mutual communication between breast cancer and its inflammatory environment is a wonderful partnership among cells fighting for genome stabilization and apoptotic death of tumor. 7. In breast cancers, there is no resistance to genotoxic or immune blocker therapies, but rather, the nonresponsive tumor cells exhaust all compensatory possibilities against therapeutic damages. **Conclusions:** Understanding the behavior and ambition of breast cancer cells may achieve a turn in therapy via applying supportive care instead of genotoxic measures.

## 1. Introduction

Breast cancer constitutes a major public health problem globally, as it is the most commonly diagnosed cancer in the world. The recent global cancer burden data estimate that there were nearly 2.3 million diagnosed breast cancer cases in 2020 and this disease is the leading cause of cancer mortality worldwide among women [1]. The therapy of breast cancer is a great challenge even today, illuminating that there are serious shortcomings in our preventive and therapeutic measures.

Breast cancer incidence and mortality exhibit a rising trend globally among both premenopausal and postmenopausal women [2]. Incidence trends for 1998–2012 were assessed by calculating the annual average percent change. In 2018, 645,000 premenopausal and 1.4 million postmenopausal breast cancer cases were diagnosed worldwide, with more than 130,000 and 490,000 breast cancer-related deaths occurring in the pre- and postmenopausal groups, respectively. Proportionally, countries with a low human development index (HDI) faced a higher burden of premenopausal breast cancer concerning both incidence and mortality as compared with higher-income countries. Surprisingly, countries with a very high HDI had the highest premenopausal and postmenopausal breast cancer incidence: 30 and 253 cases per 100,000, respectively. By contrast, countries with low and medium HDI showed the highest mortality among both premenopausal (8/100,000) and postmenopausal (53/100,000) breast cancer cases.

In five continents, country-specific data and trends of breast cancer incidence and mortality were analyzed up to 2018 [3]. Globally, 2,088,849 new breast cancer cases and 626,679 breast cancer-associated deaths were registered in 2018. The average age-standardized rate (ASR) of breast cancer incidence was 46.3 per 100,000 population globally. Examining the breast cancer incidence rate of various geographic regions, a near fourfold difference was revealed between countries exhibiting the highest and lowest ASR values (Table 1). Considering that regions showing the highest ASR values are positioned near to the Arctic or Antarctic pole, the deficient light exposure of their inhabitants emerges as strong risk factor for breast cancer. Daylight helps the maintenance of hormonal and metabolic equilibrium in human populations, while light deficiency maybe deteriorative for genomic functions leading to cancer initiation.

The breast cancer-related age-standardized mortality rate was 16.3 per 100,000 population globally and varied by three-fold among different geographic regions [3].The highest ASRs of breast cancer mortality were found in Melanesia, while the lowest death rates were reported in Eastern Asia (Table 2).The mortality of breast cancer was higher in countries with low or medium human development indices (HDIs) as compared with those with high or very high HDIs. The lower rate of breast cancer-related mortality in countries having higher HDI justifies that good economic support for public health may compensate for the geographically defined increased breast cancer incidence.

The global increasing trend in breast cancer mortality underlines that the widespread introduction of early diagnosis via mammographic screening could not keep the most frequently diagnosed cancer in women under control. In a large prospective study including more than 100,000 women with ductal carcinoma in situ (DCIS) diagnosis, it was established that DCIS carries a higher risk of breast cancer-related death than previously thought [4].Women diagnosed with DCIS are twice as likely to die as compared with the general female population in US. The high risk for invasive breast cancer development from DCIS suggests that our standard surgical and adjuvant therapeutic measures require rethinking, as they are inappropriate for ensuring a tumor-free life [5].

The presented study illuminates the crucial role of breast cancer studies in the progress of overall cancer research. Understanding the unique sensitivity of female breasts to genomic imbalance and the recognition of DNA damage-repairing activity in breast cancer cells justify that our currently used modern strategies targeting the DNA damage responses of tumors are erroneous.

## 2. Historical Survey of Omitted Opportunities for Understanding Breast Cancer Behavior

At the end of the 19th century, pulsation of breast cancers was observed parallel with the menstrual cycles of premenopausal patients. Misunderstanding of this observation led to the erroneous idea of estrogen withdrawal as a breast cancer therapy via oophorectomy [6]. Later, further results were published on achieving a tumor regression rate of <30% in breast cancer patients via therapeutic oophorectomy [7]. From that time onwards, estrogen withdrawal as a means for breast cancer prevention and therapy has become a fundamental medical principle defining a unique pathway for breast cancer care until now.

In the early 1950s, menopausal hormone therapy (MHT) became widely used among menopausal women. Synthetic estrogen, ethinylestradiol (EE), and CEE extracted from pregnant equine urine were prescribed for the care of menopausal complaints and for prophylaxis of cardiovascular diseases and thromboembolic complications [8]. In MHT user women, increased risks of cardiovascular and pulmonary thromboembolic events and of breast tumors were established [9]. Concerning the risks and benefits of synthetic and natural hormone treatments, there were no comparative studies.

In 1960, the discovery of estrogen receptor (ER) was a milestone in medical research [10]. Estrogen-binding proteins proved to be nuclear transcription factors inducing DNA replication and protein synthesis in the targeted tissues. From that time onwards, molecular studies tried to find causal correlations between excessive estrogen signaling and breast cancer induction [11,12,13,14].

Synthetic estrogen, ethinylestradiol, became the standard estrogenic component of oral contraceptive (OC) pills developed in the 1960s [15]. In OC users, thromboembolic diseases and increased risk for some cancers, including tumors of the female breast, were rare but serious complications [16,17]. Moreover, OC use increased the risk for glucose intolerance and type 2 diabetes as the chemically modified synthetic estrogen proved to be an endocrine disruptor [18]. In laboratory investigations, ethinylestradiol proved to be a histone-modifying genotoxic compound [19].

In the early 1970s, antiestrogen compounds were developed for ER positive breast cancer therapy. Blockers of liganded ER activation and aromatase enzyme inhibitors were introduced so as to spare tumors from excessive estrogen signaling [20]. However, liganded estrogen signaling is the fundamental regulator of mammalian cells, and its blockade is genotoxic, provoking desperate counteractions in both patients and their tumors [21].

The initial enthusiasm rapidly turned into frustration as about 50% of even the targeted breast tumors with ER expression proved to be non-responders to endocrine therapy. It was nominated as primary resistance [22]. Moreover, following long-term therapy, all patients showing previously good tumor responsesturned to secondary endocrine resistance, resulting in metastatic tumor spread and lethal outcome. Studies on ER-positive tumors responsive to endocrine therapy mistakenly suggested that the overexpression of ERs is an effort for survival [23], while in non-responsive tumors, the increased expression of growth factor receptors is the survival technique [24].

Extragonadal estrogen synthesis in adipose tissue was first described in 1974 [25]. In adipocytes, androgen hormones were converted to estrogens via aromatase enzyme activity. The crucial role of estrogen signals in the regulatory functions of adipose tissue was clarified much later [26]. Local aromatase activity, parallel with high estrogen concentration in mammary adipose tissue, was erroneously evaluated as fuel for the initiation and proliferation of breast cancer [27].

Breast cancers responsive to endocrine therapy successfully counteract the endocrine disruption via overexpression of ERs and restoring estrogen signaling [28]. Conversely, when the liganded activation of ERs is completely blocked in tumors, growth factor receptor overexpression desperately targets ER activation via an unliganded pathway. Non-responsive breast cancers are not fighting for their survival against antiestrogen therapy, but rather, they are incapable of counteracting the endocrine blockade.

In the 1990s, correlations between obesity and increased risk for breast cancer werereported in postmenopausal women [29]. Later, a controversial relationship was established between obesity and breast cancer, defined by the menopausal status [30]. In obese postmenopausal women, the increased aromatase activity and estrogen synthesis of abundant adipose tissue was mistakenly regarded as a causal factor of increased breast cancer risk. By contrast, in premenopausal patients, the obesity-associated hormonal defects and amenorrhea seemed to be protective for female breasts. In reality, in obese postmenopausal women, estrogen loss and the associated insulin resistance induce breast cancer development. Conversely, in obese, cycling premenopausal cases, a moderately declining estrogen signal may counteract the obesity-associated insulin resistance providing protection for the female breast [31].

In 1992, a report on a case–control study established that compensatory hyperinsulinemia in insulin-resistant cases is a significant risk factor for breast cancer independent of general adiposity or body fat distribution [32]. Analyzing the role of insulin in breast cancer development, its complex interaction with estrogen was described [33]. Insulin increases aromatase synthesis in mammary adipose cells and ER expression in tumor cells, presumably supporting the development of estrogen-dependent breast cancer. In reality, insulin resistance develops as a consequence of defective estrogen signaling [34]. Compensatory hyperinsulinemia tries to restore genome stabilization via upregulation of estrogen signaling in both healthy breast cells and breast cancers [35].

Breast cancer incidence and progression show strong disparities between African American (AA) and white American (WA) women. Among AA women, breast cancers exhibit a higher prevalence; they are more often diagnosed in young age [36] and the tumor progression is rapid, exhibiting higher aggressivity as compared with white women [37]. Ethnic/racial differences in breast cancer incidence and mortality justify that excessive pigmentation in AA women results in a relative lightdeficiency inducing estrogen deficiency, insulin resistance, hypothyroidism, and low vitaminD levels. This hormonal and metabolic imbalance increases the incidence and mortality of breast cancer [38]. The continuous growth of the black–white breast cancer mortality gap justifies that the current approaches to eliminating racial/ethnic disparities in breast cancer are not appropriate [39].

Studying the receptor landscape of breast cancers, ER-positive and ER-negative tumors were sharply separated [40]. Increased ER expression was regarded as a survival technique of estrogen-dependent tumors, whilst the ER-negative ones were kept entirely hormone-independent. Considering the high prevalence of ER-positive breast tumors in postmenopausal women, increased ER expression indicates a compensatory action asking for estrogen from the estrogen-deficient milieu [41]. Conversely, the lack of hormone receptors in breast tumors reflects the serious defect of estrogen signaling attributed to the inherited or acquired defect of ER function.

The discovery of the *BRCA1* [42] and *BRCA2* [43] genes proved to be a breakthrough in cancer research. Germline mutation of these genome safeguarding genes resulted in genomic instability and a high risk for cancer, specifically for breasts and ovaries in women. Nevertheless, appropriate BRCA proteins play essential roles in all human cell types, ensuring genomic stability [44,45].

Molecular investigations on tumor cells with the *BRCA* mutation revealed the weakness of hormonal activation of ERs, while increased compensatory unliganded ER activation was also experienced [46]. In patients with *BRCA* gene mutation, compensatory increased aromatase enzyme expression and activation as well as elevated estrogen synthesis were experienced. These findings mistakenly justified the presumed estrogen-induced genomic instability [47]. Breast cancers exhibiting *BRCA* gene mutation are predominantly basal-type ER-negative, HER2 overexpressed, and triple-negative breast cancers (TNBCs) [48,49].

Tumors with the *BRCA* gene defect may not be under excessive estrogen activation, as they lose their liganded ER activation and ER expression. In *BRCA* gene mutation carriers, the defect of liganded ER activation clearly shows a causal correlation with genomic instability and high risk for breast cancer [50].

Studies on healthy and cancer cells with *BRCA1/2* mutations have undoubtedly revealed the hard work for compensatory upregulation of estrogen signaling and genome stabilization via somatic mutations [51]. These observations suggest that somatic mutations in healthy cells try to maintain genomic stability, while in tumor cells, constitutive mutations serve the inhibition of further genomic damages rather than stimulating the proliferation.

In the early 2000s, investigations on the liganded and unliganded activation of ERs fairly promoted the development of human genetics and understanding of the rules of the whole genomic machinery [52,53]. The estradiol-regulated signaling network maintains genomic integrity and drives DNA damage repair in both men and women. Recently, the vision of E2 as a pro-carcinogenic hormone seems to be somewhat questionable [54].

In 2004, a large Women’s Health Initiative (WHI) study reported on a strikingly decreased risk of breast cancer in CEE (Premarin)-treated women compared to the placebo control cases [55]. The authors did not believe in their unusual findings and suggested strengthening investigations. CEE treatment alone without synthetic progestin was capable of genome stabilization, improving the health advantage of postmenopausal women [56].

The WHI study, resulting in breast cancer prevention by Premarin alone, was repeated several times on surviving women [57,58,59]. The genome safeguarding effect was justified in the Premarin-treated group in all repeated studies. In 2019, a global meta-analysis study was published on the worldwide epidemiological evidence of MHT-induced breast cancer, associated with all hormone formulas used [60]. Nevertheless, WHI studies justifying the breast cancer prophylaxis by Premarin treatment were not included in this examination.

Despite our developing knowledge concerning the complex roles of estrogen signaling in the genomic regulation and health maintenance of mammalians, breast cancer has been considered an estrogen-induced disease until now [61].

## 3. Estrogen Deficiency and ER Resistance Proved to Be Newly Recognized Cancer Risk Factors

In 2007, estrogen deficiency was revealed as a risk for oral cancer based on the findings of a Hungarian clinical–epidemiological study [62]. Age- and gender-related analysis of oral cancer cases revealed that men are increasingly affected by this aggressive tumor during their whole life, attributed to their smoking and drinking habits. By contrast, women in their premenopausal period are nearly completely protected from this disease. Above 50, a steeply increasing oral cancer incidence may be observed among non-smoking, non-drinking older women, parallel with their estrogen loss.

Estrogen deficiency emerged as a risk factor for further malignancies, and the principle of “estrogen induced breast cancer” became fairly questionable. Since genomic instability may not develop via quite opposite pathways at different sites, the defect of estrogen signaling emerged as a risk factor even for breast cancer [63]. In postmenopausal women, estrogen loss maybe an obvious risk factor for breast cancer as compared with premenopausal patients. In premenopausal patients, inherited or acquired gene mutations may result in weak estrogen signaling, genetic instability, and increased breast cancer risk. Among breast cancer cases, about 80% are above 50, being predominantly postmenopausal, while only 20% of them are younger, premenopausal women [64].

Thorough investigations could not find direct correlations between increased serum estrogen levels and breast cancer development in either young or postmenopausal breast cancer cases [65,66]. Epidemiological studies suggested that the better the reproductive capacity of a woman, the lower her breast cancer risk. In premenopausal women having healthy ovulatory cycles, the breast cancer risk is much lower as compared with postmenopausal cases with extremely low serum estrogen concentrations [67]. A strikingly decreased breast cancer risk may be observed in correlation with parity and, particularly, multiparity as compared with nulliparous women [68]. Conversely, anovulatory disorders and nulliparity increase the risk for breast tumors and further female cancers [69,70]. These epidemiological studies support the pathogenic role of defective estrogen signaling in breast cancer development.

Immunohistochemical imaging of estrogen receptors (ERs), progesterone receptors (PRs), and human epidermal growth factor receptors (HER2s) in breast cancers provided further possibilities to clarify the risk factors and therapeutic possibilities of variously differentiated tumors [40]. In postmenopausal breast cancer cases, tumors predominantly showed high differentiation with abundant ER expression, compensating for the decreasing level of serum estrogen [71]. There was an erroneous concept suggesting that the hormone dependence of ER positive tumors and estrogen seemed to be a fuel for their growth and metastatic spread. In reality, in tumors, a compensatory increased ER expression upregulates estrogen signaling and helps DNA stabilization in an estrogen-deficient milieu [41].

In contrast, premenopausal patients with breast cancer have predominantly poorly differentiated ER- PR- negative or TNBC-type tumors, while their serum estrogen levels maybe normal or compensatory increased, which is attributed to the defect of ERs [72]. In young patients, the predominance of poorly differentiated, hormone receptor-negative tumors may be associated with germline or acquired weakness of their liganded ER activation [41].

Germline *BRCA* gene mutation carriers exhibit a high risk for both ER-negative and TNBC-type tumors in correlation with their defect of ER expression and activation. Female breasts require balanced ER activation via liganded and unliganded pathways; consequently, they are uniquely vulnerable to the fault of estrogen signaling in *BRCA* gene mutation carriers. In young women with weak estrogen signaling and ER-negative breast cancer, a deceptive high estrogen level may be experienced, associated with clinical signs of menstrual disorders and anovulatory infertility [73].

In conclusion, either estrogen deficiency or defective liganded ER activation may lead to genomic instability, increasing the risk for breast cancer.

## 4. Genes Activated by Estrogen Upregulate DNA Stabilization and Silence Cell Proliferation in Breast Cancer Cells

Estrogen liganded ERs are transcriptional factors regulating the genomic machinery as hubs in the network of regulatory circuits. DNA stabilization, cell proliferation/silencing and fuel supply are the main regulatory circuits of ERs, orchestrating all genomic processes in mammalian cells [74].

The *DNA stabilization circuit* of ERs enjoys primacy over all other regulatory functions and requires continuous estrogen activation of ERs. Three regulatory proteins, ER alpha, BRCA1, and aromatase enzyme (A450), create a signaling network via triangular partnership. Liganded ER-alpha is capable of occupying the promoter regions of *ESR1*, *BRCA1*, and *CYP19A* aromatase genes and driving messenger RNA (mRNA) expressions. The next step is the expression of ER-alpha, BRCA1 protein, and aromatase enzyme via translation. Abundant aromatase expression increases estrogen synthesis and the newly formed estrogen activates ERs again, closing the regulatory circuit. Estrogen parallelly drives the genome stabilizer circuit and estrogen signaling according to the requirements. Decreased estrogen supply or weakness of any of the three regulatory proteins endangers genomic stability and promotes emergency measures.

In the *cell proliferation circuit*, liganded ERs regulate and supervise the rapidity and quality of cell proliferation via upregulation or downregulation, adapting to the necessities. ERs exhibit mutual interaction with membrane-associated growth factor receptors (GFRs). The expression and activation of growth factors (GFs) and their receptors (GFRs) are also regulated under estrogen control. Growth factor signal (GFS) transduction upregulates kinase cascades via PI3-K/AKT/mTOR and RAS/RAF/MEK/MAPK pathways. Kinase cascades mediate nuclear ER activation via the unliganded pathway, facilitating further expression of specific genes.

Through the *fuel supply circuit*, estrogen-liganded ERs regulate all phases of glucose uptake and orchestrate all players participating in glucose homeostasis. Estrogen-activated genes drive insulin synthesis in the pancreatic islands and the expression of insulin receptors (IRs) in all human cells. Liganded ERs stimulate glucose transporter 4 (GLUT4) gene expressions and GLUT4 translocation to the cell membrane so as to mediate glucose uptake. Plasma membrane-associated ERs activate insulin receptor substrate 1 (IRS-1), stimulating the kinase cascade through the PI3-K/AKT/mTOR pathway. Kinase cascade confers unliganded activation for nuclear ERs, closing the regulatory circuit and upgrading glucose uptake.

Genomic instability is a crisis situation even for cancer cells, moving the activation of available DNA-protecting mechanisms [51]. Breast cancer cells use and strengthen their genome stabilizer pathways via gene amplifications and activating mutations. Highly differentiated breast tumors show increased expression of ERs and estrogen synthesis driving liganded ER activation and genome stabilization.In moderately differentiated breast cancers, the loss of hormone receptors upregulates the expression of HER2 receptors and kinase cascade members, strengthening the unliganded activation of scarcely occurring ERs. In triple-receptor-negative, poorly differentiated tumors, there is no possibility for either liganded or unliganded ER activation, resulting in unrestrained proliferation.

## 5. In Breast Cancer Cells, Estrogen Upregulates the Genome Stabilizer Circuit Counteracting Pro-Oncogenic Processes

In tumor cells, estradiol treatment *stimulates the liganded ER activation*. Four types of estrogens, estrone, estradiol, estriol, and estetrol, significantly increase the ERexpression of ER-positive breast cancer cell lines as compared with the untreated controls [75]. This activation of ERs is an effort for the upregulation of genome stabilization and stopping cell proliferation, rather than a survival technique.

In breast cancer cells, estrogen treatment facilitates the expression of membrane associated growth factor receptors, both EGFR and HER2, *upregulating even the unliganded activation of ERs* [27]. In addition, estradiol treatment enhances the activity of phosphatidylinositol 3-kinase (PI 3-K)/Akt system in tumor cells [76]. Estrogen promotes the activation of membrane-associated growth factor receptors (GFRs) and kinase cascades serving DNA stabilization through increased unliganded ER activation [24]. In tumor cells, crosstalk between overexpressed ERs and GFRs targets the improvement of genomic stability, leading to apoptotic death.

In breast tumor cells, estrogen treatment frequently induces an *amplification of ESR1 gene* at 6q25 locus upregulating ER protein synthesis [77]. During breast cancer treatment with estrogen, a cluster of non-coding RNAs is experienced, activating the appropriate *ESR1* locus [78]. A longer disease-free survival is experienced in patients showing *ESR1* gene amplification in breast tumors compared to those without this alteration [79]. These clinical observations justify that, in tumors, an estrogen-induced amplification of the *ESR1* gene is not a pro-oncogenic adaptation, but rather, an abundant expression of ERs may facilitate tumor responses.

In estradiol-treated tumor cells, activated ERs confer the transcription and an *increased expression of lncRNAs*, such as HOTAIR [80]. Increased HOTAIR expression induces epigenetic changes on both *ESR1* and *BRCA1* genes, promoting increased expression of ER-alpha, strengthening DNA stabilization and tumor regression [81]. Increased HOTAIR expression in tumors of breast cancer cases is associated with a lower risk of relapse and mortality compared to those having low expression of HOTAIR in their cancers [82].

In breast tumor cells, estrogen activation of ERs is capable of *increasing aromatase enzyme expression and estrogen synthesis*, facilitating the liganded activation of ERs. ER-negative tumor cells were transfected with exogenous ER alpha, and estradiol treatment enhanced the aromatase activity in a dose-dependent manner [83]. In breast cancer cells, estradiol may enhance the expression of aromatase enzyme through ER-alpha activation and increase aromatase activity via enhanced tyrosine phosphorylation of the enzyme [84].

High *aromatase activity and an increased* in situ *estrogen concentration* were experienced at the invasive front of breast cancers, where interaction between the tumor and neighboring tissues may define the expansion or regression of cancer [85]. Aromatase activity of surgically removed tumor samples and patients’ survival time after surgery showed a direct correlation in breast cancer cases [86,87].

In the MCF-7 breast cancer cell line, estrogen treatment stimulated *increased* BRCA1 *protein expression* [88]. In turn, the BRCA1 protein promoted *ESR1*-gene activation, leading to an increased expression of ER-alpha mRNA and ER protein in breast cancer cell lines [89]. In hypoxia, which is an important factor in solid tumor progression, a decrease of both BRCA1 and ERα expression was demonstrated in MCF-7 cells [90]. These observations support that ligand-activated ER alpha and BRCA1 protein together upregulate the circuit of genome stabilization, leading to self-directed death of tumor cells [50].

In tumor cells, estradiol treatment *increases insulin-assisted glucose uptake*. In MCF-7 tumor cell lines, estradiol facilitates insulin signaling through an increased expression of insulin receptor substrate-1 (IRS-1) [91]. In MCF-7 cell lines, estradiol helps in the entrance of glucose through the cell membrane via increasing GLUT4 expression. Estradiol treatment increases PI3K/Akt kinase cascade activation, helping in the translocation of GLUT4 into the plasma membrane driven by ER-alpha [92]. These results reveal that estrogen increases insulin-assisted glucose uptake even into cancer cells, ensuring energy for the improvement of DNA stability, rather than increasing proliferative activity.

## 6. Molecular Classification of Breast Cancer Subtypes Is a Mirror Reflecting the Defect of Estrogen Signaling and Genomic Damage in Patients

Breast cancer is apparently a multifaceted disease originating from the excessive proliferation of either lobular or ductal breast epithelium [93]. The molecular profile is the most important factor for breast cancer categorization [94]. The molecular heterogeneity of breast cancers was exposed through the expression of various gene panels. Four main groups of breast cancers were established: luminal A, luminal B, HER2 overexpression, and basal-like triple-negative breast cancers (TNBCs). Moreover, a highly differentiated subgroup of breast cancer was also described morphologically, resembling the tumors of luminal A group, but it is associated with a poor outcome of the disease.

Breast cancer subgroups are recognized by immunohistochemical staining of receptors. In clinical practice, ER-alpha, progesterone (PR), and human epidermal growth factor receptor (HER2) expressions are evaluated [93]. The increased expression of certain receptor groups in tumors is erroneously evaluated in an aggressive effort for survival, leading to unrestrained proliferation. According to current therapeutic principles, the targeted inhibition of highly expressed receptors, such as ERs or HER2 proteins, may inhibit the altered DNA damage response of tumors. Conversely, in tumors, the overexpression of certain receptors reflects the upregulation of pathways, serving the improvement of defective genomic processes and DNA repair [81].

In breast cancer cells, a lack or low expression of certain receptors is associated with aggressive growth and, at the same time, reveals the critical points of serious genomic defects which need to be repaired [41]. Currently, poorly differentiated, basal-type, triple-negative breast cancers are treated with chemotherapy or immune therapy as they do not have receptors to be targeted [95].

*Luminal A subtype of breast cancers* are highly differentiated tumors exhibiting increased expression of hormone receptors, ER alpha, and progesterone receptor (PR), and they show good prognosis. In breast cancer, increased ER expression is erroneously regarded as a pivotal player in tumor initiation and growth, conferring excessive estrogen signaling. In clinical practice, ER expression in breast cancer is a prerequisite for the efficacy of antiestrogen treatment; however, nearly half of ER-rich tumors prove to be endocrine-resistant [25]. In ER-positive breast cancers, *ESR1* gene amplification and point mutation are the supposed causal factors for increased aggressivity and endocrine resistance of tumors [96]. In reality, mutational changes in the *ESR1* gene are desperate efforts counteracting the endocrine disruptor therapy so as to save estrogen signaling in the absence of estrogen [28].

Various studies have published different *ESR1* amplification frequencies in breast cancers, ranging from 0% to 75% [77]. These various results show the different capacities of tumors for the genome-repairing activation of estrogen signaling. Recently, in tamoxifen-responsive, early-stage breast cancers, focal *ESR1* gene amplification was a powerful predictor for the long-term tumor-free survival of patients [97]. These results suggest that, in the early stages of tumors, somatic mutations of the *ESR1* gene may effectively facilitate genomic stabilization. Conversely, in advanced breast cancers, even extensive *ESR1* gene mutations and ER protein modifications are incapable of counteracting the excessive genomic damage.

In metastatic breast cancers, specific *ESR1* mutations have been identified to induce estrogen-independent activation of ERs. These mutations lead to constitutive activation of ERs and reduced sensitivity to ER antagonists [98]. The authors suggested the development of new ER antagonists with appropriate efficacy against certain *ESR1* mutants. In reality, the development of estrogen-independent ER activation in tumors is an ultimate refuge against the exhaustive endocrine disruptor treatment, rather than pro-oncogenic alteration.

Early diagnosis and treatment of ER-positive breast cancer does not ensure long-term, tumor-free survival for patients [5]. Tamoxifen treatment of early ER-positive tumors could result in late tumor recurrence after 10–15 years [99]. These findings highlight that antiestrogen therapy is not the correct routine to protect against the targeted ER-positive breast cancers. The source of tumor recurrence is a persistent genomic instability of the female breast rather than survival of residual tumor cells. Both endocrine treatment and chemotherapy further deteriorate the genomic defect in the targeted breast tissue, helping in new tumor initiation.

*Luminal B subtype of breast cancer* shows some dedifferentiation, and the disease has a worse prognosis as compared with luminal A-type tumors. They exhibit decreased expression of ER alpha and PR, and, in some cases, a lack of PR may be observed in association with the declining estrogen signal [100]. In certain luminal B tumors, somatic mutations of TP53 and HER2 expression may also be experienced [101]. In these tumors, somatic *TP53* mutations are not cancer driver alterations, but rather, they provide additional DNA safeguarding via changes in the p53 protein. In luminal B cancers, increasing genomic damage induces HER2 expression, strengthening ER activation via the unliganded pathway [24].

Tamoxifen therapy in women with ER-positive, PR-negative and HER2 positive tumors resulted in more frequent tumor recurrence and a higher mortality rate compared to patients without tamoxifen treatment [102]. This experience justifies that the declining ER signal in type B breast cancers is further damaged by the ER blockade. Conversely, against ER-positive, PR-negative luminal B type tumors, natural estrogen (Premarin) therapy achieved significant tumor regression and improved the survival of patients via genome stabilization [103].

ER/HER2 co-expression may presumably disturb responses to both anti-HER2–directed and endocrine therapy due to crosstalk between the ER and HER2 pathways. For type B breast cancers that are both ER and HER2 positive, a combined HER2/ER blockade has been recommended as an effective treatment strategy [104]. This double blockade on HER2 and ER may be catastrophic for patients, as in B-type tumors, these regulatory pathways together restore the altered ER signaling and genomic defects.

In the *HER2-enriched subtype of breast cancer*, the lack of ER/PR expression and overexpression of HER2 are characteristic findings. HER2-enriched breast tumors show faster growth than luminal types, and patients have poorer prognosis. The loss of ER and PR expression in HER2-enriched tumors reflects the failure of estrogen signaling and serious errors in genomic functions [24]. In ER/PR negative breast cancers, HER2 overexpression is erroneously taken for a stimulator of tumor growth, similarly to increased expression of other growth factor receptors [93]. In reality, cognition of DNA damage induces HER2 overexpression as an adaptive response serving compensatory unliganded ER activation, even when ERs are not detectable [24]. Therapies targeting the HER2 protein in these tumor types achieve ambiguous results, as only a successful counteraction may produce a transient tumor response in a minority of cases [28].

Genomic analysis of HER2-enriched breast cancer revealed higher expression of the *ERBB3* gene and lower expression of the *ESR1* gene compared to the non-HER2-enriched subtype [105]. These findings are concordant with the compensatory increased expression of the HER2 protein and the apparently missing expression of ERs in the HER2-enriched breast cancer subtype.

*The triple-negative or basal-like subtype of breast cancer* is characterized by the lack of ER, PR and HER2. Triple-negative breast cancer (TNBC) is frequently associated with germline gene mutations, genomic instability, and defective estrogen signaling [41]. TNBC type breast cancers exhibit low-grade differentiation, and they are more aggressive than any other breast cancer subtype.

In TNBC-type tumors, the missing estrogen, progesterone, and HER-2 receptors reflect severe damage to the whole genome. Clinically, TNBCs exhibit rapid growth and a poor prognosis. Self-directed DNA repair is impossible for TNBCs, as ERs seem to be absent and the liganded and non-liganded activation of ERs are missing [41]. Development of a TNBC-type tumor in women indicates seriously defective estrogen signaling. The increased prevalence of TNBCs among black American women may be associated with light deficiency-associated hormonal disorders, as dark pigmentation is incongruent with the light-deficient northern milieu [38].

Basal-type hormone receptor-negative and TNBC-type breast cancers with serious genomic damage are regarded as being refractory to antiestrogen therapy. Chemotherapy remains the standard for TNBC treatment; however, new drug-delivery methods are compulsory as the signaling pathways within these tumors are not clarified [95]. Since hormone receptor-negative breast tumors are “immune hot”, showing strong inflammatory infiltration, immune checkpoint inhibitors (ICPis) were introduced, targeting the presumably pro-oncogenic immune competent cells [106]. Despite the promising results of IPCi therapy, these drugs may induce immune-related endocrine dysfunction that could lead to life-threatening complications. The authors suggest continuing IPCi therapy, as the adverse endocrine complications may be treated with hormone replacement.

In conclusion, molecular classification of breast tumor subtypes is a mirror reflecting the defect of estrogen signaling and genomic damage in cancer patients. In women, robust estrogen signaling may repress breast cancer initiation, and the accidentally developing tumors are highly ER-positive in these cases. By contrast, in women with weak estrogen signaling, the associated genomic instability liberates breast cancer development, and their tumors are predominantly aggressive, hormone receptor-negative, or triple-negative breast cancers.

## 7. Dynamic Communication between Breast Cancers and Their Microenvironment during Anticancer Fight

Since the 2000s, the microenvironment of cancers has been an essential participant in tumor growth and aggressive metastatic extension [107]. According to recent aspects, cancer is a combined lesion comprising a tumor lump and an altered cellular microenvironment [108,109]. Tumor and cellular infiltration of the microenvironment exhibit active communication, mistakenly suggesting collaboration and serving unrestrained tumor invasion as well as escape from immune reactions and anticancer therapies.

Highly elevated expression of certain regulatory proteins and signaling molecules in cancers and in their neighboring cellular environment are mistakenly regarded as pro-oncogenic factors strengthening the concept of pro-tumorigenic conspiration [109,110,111]. Moreover, when genes with roles in genome stabilization are amplified or altered by mutations, they are regarded as pro-oncogenic changes instead of self-directed labors for genomic repair [96,112,113,114,115]. In breast cancers, the upregulation of estrogen synthesis and ER activation via various pathways are taken for crucial conditions helping tumor growth. Conversely, in cancers, intensifying signaling pathways and the accumulation of somatic mutations do not product cancer-driving factors, but rather they facilitate estrogen signaling, DNA repair, and apoptotic death [81].

In solid tumors, cancer-associated fibroblasts (CAFs) are the major players in the tumor microenvironment [108]. In peritumoral cellular infiltration, CAFs and all kinds of immune-proficient cells are in mutual connection with tumor cells [109]. Membrane-bordered extracellular vesicles (EVs) carry intercellular messages comprising different signaling molecules, regulatory proteins, and nucleotide templates. Intercellular communication between tumors and their microenvironment ismistakenly regarded as help for tumor growth and invasion.

CAF secretome frequently comprises growth factors and growth factor receptors. Growth factor signaling cascade is regarded as pro-tumorigenic stimulus upregulating tumor growth and invasion [116], while this regulatory pathway confers the possibility for unliganded ER activation.

Cytokine secretion of CAFs, monocytes, and macrophages regulates the inflammatory and immunological reactions in the peritumoral region [117]. Pro-inflammatory cytokines recruit inflammatory immune competent cells and activate aromatase expression, leading to increased estrogen synthesis [118]. An appropriate estrogen concentration is not a cancer driver factor, but rather, it orchestrates the anticancer combat of immune-competent cells. When the fight is successful, anti-inflammatory cytokines alleviate inflammatory response and allow the estrogen concentration to decrease [119].

Numerous tumors induce increased aromatase synthesis in both tumor cells and peritumoral stroma via high proinflammatory cytokine secretion [120]. In breast cancers, abundant expression of aromatase has been observed in tumor cells, in fibrous cells, and in adjacent adipocytes, suggesting a pro-carcinogenic activity of increased estrogen synthesis [121]. Conversely, a parallel clinical and genetic investigation on breast cancer cases justified a significant correlation between missing aromatase levels in surgically removed tumors and high local recurrence rates [87]. In conclusion, there is no causal correlation between a high estrogen concentration and rapid tumor growth, but rather, the lack of estrogen production in tumors is linked with poor prognosis of the disease.

Recently, mammary adipocytes have been particularly blamed for the growth and invasion of breast cancer, since the female breast comprises abundant fatty tissue. Cancer-associated adipocytes (CAAs) are specific players in the microenvironment of breast tumors, presumably helping in progression and metastatic spread [122]. Activated CAAs show dedifferentiation, and their copious secretome comprises different cytokines and adipokines. CAAs comprise tiny lipid droplets serving the rapid energy supply of adjacent tumor cells. They participate in remodeling the extracellular matrix, increasing aromatase expression, and modulating the immune cell functions. In reality, activated CAAs serve the anticancer fight via adipokine secretion, energy supply, and regulatory commands for all neighboring cells similarly, like activated CAFs.

In summary, cancers and their peritumoral cellular infiltration are allies in the fight against DNA damage and uncontrolled proliferation of tumors. The graver the DNA damage in tumors, the stronger the cellular infiltration of microenvironment. The aim is the restoration of DNA stability, compelling apoptotic death. Intensive peritumoral cellular infiltration coupled with invasive tumor growth may not be regarded as a victory of conspirator partners, but rather, it is a collective defeat of cancer and its microenvironment.

## 8. Correlations between Breast Cancer Subtypes and the Characteristics of Their Cellular Microenvironment

Recently, correlations between the receptor status of breast cancers and peritumoral inflammatory reaction have been thoroughly investigated. Abundant immune cell infiltration was experienced around “immune-hot” hormone receptor-negative breast cancers with a predominance of B cells, Th1 T cells, and cytotoxic T lymphocytes (CTLs), while ER-positive cancers proved to be immune-cold [123].

**Figure 1:**
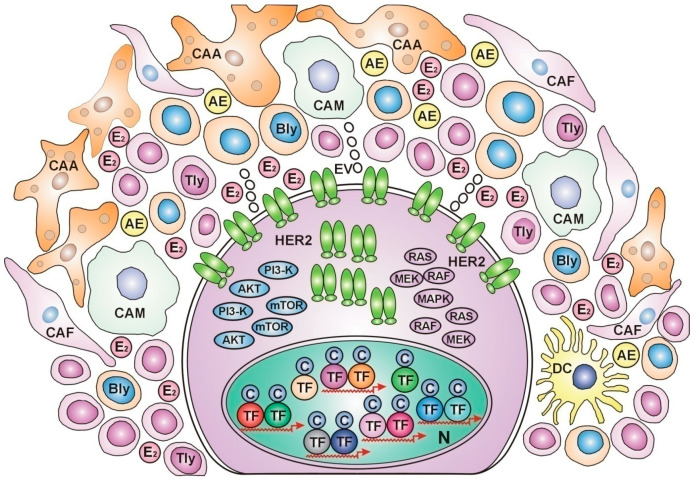
HER2-overexpressed, immune-hot breast cancer cell *In the nucleus*, numerous transcriptional factors (TFs) and their cofactors (Cs) work on the expression of various genes without estrogen regulation. Estrogen receptors are undetectable. *In the cytoplasm*, intensive protein synthesis produces HER2 and the members of its kinase cascades: PI3-K, AKT, mTOR, RAS, RAF, MEK, and MAPK, for rectifying cell proliferation via unliganded ER activation. *At the cell membrane*, numerous HER2 receptors wait to find ERs appropriate for activation and improving genomic damages. *In the peritumoral ring*, cancer-associated adipocytes (CAAs), cancer-associated fibroblasts (CAFs), and dense immune cell infiltration help in the genomic repair and apoptosis of tumor cells. The cytokine secretome promotes aromatase enzyme (AE) expression and estradiol (E_2_) synthesis. CAM: cancer-associated monocyte, Bly: b lymphocyte, TLy: t lymphocyte, DC: dendritic cell, EV: extracellular vesicle, N: nucleus.

Extensive analysis was conducted on the features of the tumor microenvironment in each breast cancer subtype [124]. Basal-like and HER2-enriched tumors were associated with high immune scores, expressing the most immune regulatory targets and abundant immune cell infiltration. These poorly differentiated breast cancer subtypes could be defined as “immune-hot” tumors (Figure 1)

**Figure 2:**
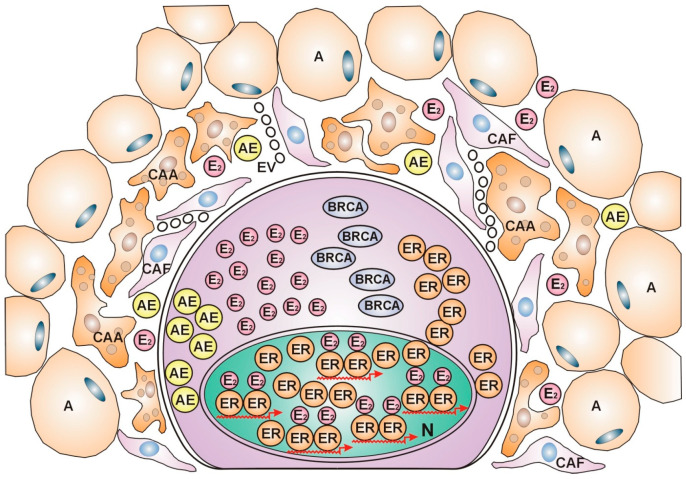
ER-positive, immune-cold breast cancer cell. *The nucleus is* crowded with estrogen (E_2_)-activated estrogen receptors (ERs), showing busy transcriptional activity. *In the cytoplasm*, intensive protein synthesis produces the players in the genome stabilizer circuit: estrogen receptors (ERs), BRCA proteins, and aromatase enzyme (AE), completed with estrogen (E_2_) synthesis. *In the peritumoral ring*, lipid-laden adipocytes (As) border the actively helping cells, cancer-associated adipocytes (CAAs), and cancer-associated fibroblasts (CAFs). The cytokine secretome activates aromatase enzyme synthesis.

In contrast, luminal A and luminal B subtypes showing ER expression are associated with low immune scores and weak immune cell infiltration, suggesting that these subtypes may be regarded as “immune-cold” cancers [124]. Highly differentiated ER-positive tumors do not require strong immune cell support for their anticancer fight as cancer-associated fibroblasts (CAFs), and cancer-associated adipocytes (CAAs) are capable of cytokine secretion, facilitating aromatase expression and estrogen synthesis. Estrogen activation of abundant ERs upregulates estrogen signaling and DNA repair in ER-positive tumors without a dense inflammatory reaction [Figure 2].

In ER-positive breast cancer cases, gene expression analysis reveals that aromatase inhibitor (letrozole) treatment persistently increases the density of immune cells around tumors comprising subsets of B cells and T-helper lymphocytes [110]. This observation underlines that inhibition of estrogen signaling is an emergency state, even for tumors. In ER-positive, “immune-cold” breast cancers, letrozole attacks against estrogen regulation causerapid recruiting of dense immune cell infiltration and copious cytokine secretion, upregulating aromatase expression and estrogen synthesis.

In conclusion, hormone receptor-positive tumors are capable of fighting for increased aromatase expression and estrogen synthesis with the assistance of bordering CAFs and CAMs, aiming at genomic repair. By contrast, hormone receptor-negative and TNBC-type cancers, as well as tumors exhaustively treated by antiestrogens, are in an emergency condition, requiring the help of immune-competent cells.

## 9. Animal Models and Their Application in Breast Cancer Research

Animal models are applied for studies on the biological behavior of breast cancer and for preclinical investigations of response or resistance mechanisms of tumors under newly developed therapies [125].

The physiology of mammalians and their tumorigenesis in the breasts are similar to those of humans. Rodents are the preferred animals for breast cancer research, mice and rats in particular. There are troubling differences in mice compared with humans, as they tolerate higher drug doses without toxic complications. In addition, in mice, the metastasis of breast cancer generally occurs in the lung, while human breast tumors have metastases to the lymph nodes, bone, liver, and brain [126].

Spontaneous breast tumors may frequently occur naturally in rodents. Animal models with spontaneous breast cancer are appropriate for investigation on cancer etiology and epidemiology. In the case of induced animal models, scientists provoke tumorigenesis via application of chemical, physical, or biological carcinogens [127]. In mice, the majority of chemically provoked breast cancers are B subtypes [128]. N-methyl-N-nitrosourea (MNU) NMU-induced primary rat tumors are similar to ERα-positive low-grade human breast cancer [129]. In rats, dimethyl-benz-anthracene (DMBA) administration resulted in near 100% breast tumor development within three months [130]. DMBA and medroxyprogesterone acetate (MPA) treatment induced breast carcinoma in tree shrews in correlation with the activation of Akt pathway via *PIK3CA* gene mutation [131]. These molecular changes are rather compensatory efforts for unliganded ER activation instead of pro-oncogenic stimuli. In animals, biological breast cancer induction may be performed by lentiviruses via overexpressing oncogenes or silencing cancer-suppressor genes [132].

Transplantation of spontaneous or induced breast tumors yields transplantation animal models. The most liked transplantation model is the human xenograft in animals, which is appropriate for testing new therapies [133]. Hungarian authors introduced a *BRCA1*−/−, *p53*−/−breast tumor cell line in mice (CST) with similar features and therapeutic sensitivity to TNBC-type human tumors in *BRCA* mutation carriers [134].

Cell-derived xenografts (CDX) originating from human tumor cell lines may be inoculated subcutaneously, intravenously, and cardially into immunodeficient mice lacking T cells, B cells, NK cells, and macrophages in various combinations [135]. MCF-7 and T47D cell lines are luminal A type and ER-positive. Cell lines with features of HER2 subtypes have low tumorigenic capacity, while TNBC-like cell lines show high tumorigenic activity [136].

Patient-derived xenografts (PDXs) derive directly from the tumor tissue of patients gene expression profiles, metastatic capacity, and drug responses in animal models to their surgically removed counterparts [137]. PDX-bearing animal models forecast the outcome of the disease, aid in preclinical drug evaluation, and may be used for personalized medicine [138].

A humanized PDX model (Hu-PDX) was developed by rebuilding the human immune system in immunodeficient mice via intravenously injected immune-competent human cells [139]. This model may authentically mimic the microenvironment of human cancers and is suitable for the investigation of tumor immunology.

Genetically engineered animal models (GEMMs) for breast cancer studies maybe created via transgenic technology [140]. Artificial overexpression of the presumed breast cancer-specific oncogenes, such as *HER2*/*ERBB2*, may advance breast cancer research in transgenic mice. Animal models with knockout of their tumor susceptibility genes, such as *BRCA1/2, p53*, and *PTEN*, may spontaneously develop tumors in the breast and other organs [141].

Animal models are valuable in breast cancer research, supplying important information on the initiation, progression, treatment response, and resistance mechanisms of breast cancer.

## 10. Estrogen Prevention and Therapy for Breast Cancer

Experiments on carcinogen-induced mammary tumor models have justified that pregnancy either before or soon after exposure to a chemical carcinogen is proven to be highly protective against breast cancer development in rodents [142,143,144]. Short-term treatment with high, pregnancy-mimicking levels of estradiol with or without progesterone was also highly protective against mammary carcinogenesis in rodents [145,146]. Synthetic estrogen (ethinylestradiol) plus synthetic progesterone (megesterol acetate) treatment for 3 weeks resulted in significantly lower mammary cancer incidence in rats as compared with untreated controls after 6 months of the exposure to a chemical carcinogen [147].

The mechanism of breast cancer-preventive hormonal activity was examined in genetically challenged mouse models [148]. High-dose estrogen/progesterone treatment reduced the incidence of mammary tumors by more than 60% in mice with oncogenic overexpression of the HER2/neu. Considering that growth factor overexpression is not oncogenic action, but rather an unliganded activation of weak, E_2_-refractory ERs, estrogen treatment helps in ER activation and genome stabilization [24]. Moreover, in mice with homozygous deletions of the *Tp53* gene, exogenous estrogen/progesterone treatment reduced mammary tumor development by at least 70% [148]. These results justify that hormone-mediated protection of breast tissue may also have alternate pathways independent of p53 protein activation [149].

In clinical practice, parity reduces breast cancer risk even among germline *BRCA1/2* mutation carrier women [150]. The sky-high estrogen levels in pregnancy drive the genome stabilizer circuit and the increased expression of estrogen-regulated genes, compensating for the weakness of genome-safeguarding BRCA proteins [50].

The hormone-induced breast cancer prevention achieved in animal studies has not been similarly successful in human practice. In MHT studies, the application of endocrine-disrupting synthetic estrogens and synthetic progestins resulted in deregulation of ERs, leading to an increased risk of thromboembolic complications and breast cancer [56]. Moreover, in breast cancer cases, high-dose synthetic estrogen treatment resulted in modest tumor regression and high toxicity [18]. Using Premarin in high doses seems to be risky in human practice. In contrast, the endocrine disruptor ethinylestradiol was mistakenly regarded as a bioidentical hormone appropriate for human therapy even at high doses.

WHI investigators published horse urine-derived CEE (Premarin) use alone in a great prospective, placebo-controlled MHT study, and a shocking breast cancer-preventive effect was experienced in the hormone-treated group [55]. This investigation was repeated several times on the participating women remaining alive. The long-term genome stabilizer effect of Premarin was justified by the decreased breast cancer incidence in the hormone-treated group even after 16 years [59].

In patients with ER-positive, PR-negative luminal B type tumors, Premarin treatment induced a significant reduction in cancer growth and decreased tumor-associated mortality [103]. In contrast, worsening results of tamoxifen therapy were experienced with ER/HER2-positive luminal B type tumors as compared with the untreated controls [102].

In HER2-overexpressed, apparently hormone receptor-negative tumors, E_2_ refractory ERs occur in the background, as the activated HER2 and kinase cascade pathways serve increased unliganded ER activation. High-dose estrogen treatment may upregulate the activation and expression of hidden E_2_-resistant ERs, driving the genome stabilizer circuit [151]. At the same time, high-dose estrogen silences the peritumoral immune cell infiltration, as an appropriate estrogen concentration facilitates the beneficial anticancer effects of immune competent cells.

The conviction that TNBC is a completely hormone-independent tumor is quite incorrect. Although TNBCs are apparently deficient in both hormone receptors and growth factor receptors, some hidden components of the liganded or unliganded pathways of ER activation may arise in them. Interestingly, 5–10% of ER-negative breast cancers have shown responses to tamoxifen treatment [152]. In a subtype of TNBCs, a specific isoform of estrogen receptor-alpha (ER-α36) was observed. Icaritin, a prenylflavonoid derivate, affected ER-α36-mediated pathways and tumor growth, showing a possibility for the development of a novel therapeutic agent against TNBC.

In some primary TNBC cases, arising ER, PR, or HER2 expression has been observed in metastatic lesions [153]. In a systematic review and meta-analysis, average percentages of negative-to-positive receptor conversion in metastatic tumors were 21.5% for ERs, 15.9% for PRs, and 9.5% for HER2s. Among patients with ER-negative primary tumors, conversion to a positive ER status in their metastatic lesion was associated with prolonged survival compared to those without conversion in their metastasis [154]. The appearance of ERs in the metastatic lesion of TNBC increased the possibility of successful high-dose estrogen therapy.

In tumors exhaustively treated with tamoxifen, the achieved ER blockade may be regarded as an artificially created ER-negative status, as estrogen signaling suffers irreparable damage. Among patients with tamoxifen-resistant advanced breast cancer, estrogen treatment dramatically decreases breast cancer related mortality [155]. In tumors, with tamoxifen-blocked ERs, estrogen treatment induces abundant new ER expression and drives genome stabilization. In patients with aromatase inhibitor-resistant tumors, estrogen treatment induces regression of metastatic cancers and extends survival [156]. In tumors exhaustively treated with aromatase inhibitor, therapeutic estrogen is bound by the overexpressed ERs driving the genome stabilization circuit. These findings help to reevaluate the view of estrogen-induced breast cancer and the role of antiestrogens in breast cancer therapy [157].

Medical stimulation of ER protein expression would be a promising new method for the therapy of ER-negative and TNBC-type tumors. A receptor-negative breast cancer cell line was transfected with an exogenous estrogen receptor, and the following estrogen treatment reduced the proliferative and metastatic capacity of cancer cells [158]. This experiment justified that induction of ER expression in ER-negative cancers may increase their vulnerability to estrogen therapy.

In 1989, the results of a milestone experiment were published on the induction of protein synthesis in cells by transfecting them with exogenous mRNA template. The expression of an appropriate protein was stimulated via translation [159]. Later, artificially synthesized mRNA was injected into the muscles of mice as a direct gene transfer so as to generate the expression of the desired muscular protein [160]. The authors recommended the use of mRNA technology for vaccination against pathogenic microbes. These experiments provided the earliest trials ofthe development of mRNA vaccination during the outbreak of the COVID-19 epidemic [161].

Beyond vaccination, the mRNA technology may have enormous potential in medical practice. Malone’s technology is an appropriate method for inducing expression of various transcriptional factors or enzymes in patients via a direct gene transfer technique. In genetically challenged women with decreased ER expression and/or E_2_-refractory ERs, the facilitation of ER production via *ESR1*mRNA treatment increases the expression of estrogen-regulated genes, preventing the development of cancer in the breasts and ovaries.

Preoperative *ESR1* mRNA therapy in cases with an advanced stage of ER-negative or TNBC-type breast cancer may result in an obvious tumor response coupled with stimulated immune-competent cells in the neighboring tissues. In the postoperative period, local *ESR1* mRNA therapy and the associated ER expression improve all genomic functions in the residual breast tissue, inhibiting cancer recurrence.

In conclusion, estrogen treatment is an adequate therapy for ER-positive breast tumors, whereas high estrogen doses may be efficient even against ER-negative breast cancers. In the future, the medical motivation of ER expression would be an excellent cure and secondary prevention for breast cancer patients with inherited or acquired defects of ERs.

## 11. Conclusions

Breast cancer care has developed through the centuries, from estrogen withdrawal by surgical oophorectomy to the current use of radiological and chemical targeting of estrogen signaling. There have been major inventions providing possibilities for the evaluation of estrogen’s significant role in human health, while at the same time, unfortunate failures and misunderstandings have directed breast cancer research away from the right direction.

The therapeutic use of estrogen withdrawal against breast cancer has been an erroneous route; however, the compensatory upregulation of estrogen signaling can achieve deceiving tumor responses in <30% of patients. Major mistakes may be established based on the therapeutic experiences, seriously delaying the progress of both breast cancer care and overall cancer therapy.

The first principal error is the therapeutic attack against the regulatory pathways of breast cancer cells, supposing that tumor killing is the right way to recovery. In reality, cancer cells recognize their genomic defects and show adaptive responses so as to achieve DNA repair and apoptotic death. The therapeutic blockade of DNA damage responses in breast cancer strengthens genomic failures in both tumors and patients.

The second mistake is the supposed pro-oncogenic role of accumulated somatic gene mutations and overexpression of their altered regulatory proteins in tumors, while these efforts endeavor to rectify genomic defects and facilitate apoptotic death.

The third mistake is evaluating a tumor response rate under 30% as a therapeutic success of endocrine disruptor treatment. This modest response rate may be attributed to the counteraction of breast cancer against genotoxic treatment; however, in the majority of cases, the genome-defending efforts of tumors remain unsuccessful.

The fourth mistake is the establishment of “tumor resistance” in the case of therapeutic failures. There is no resistance against genotoxic therapy, but rather, tumors may be incapable of counteracting the excessive genomic damage caused by endocrine disruptor therapy. In cancers refractory to endocrine treatment, the genomic regulation breaks down, leading to unrestrained proliferation.

Chemotherapy, radiotherapy, and excessive mutilation are methods for radical killing of even the last tumor cell, mistakenly believing that residual tumor cells cause tumor recurrence after years or decades. In reality, in cancer patients, the persisting genomic instability causes new tumor initiation in their highly vulnerable organs. The genome-damaging and immune-blocking modern therapies assist in the recurrence of tumors even after a longer period of recovery.

Understanding the efforts for DNA repair in breast cancer cells requires a turn in tumor therapy; we should help the genome stabilizer responses of breast tumors and the immune system rather than provoking additional genomic damage.

## Figures and Tables

**Table 1:** Age-standardized rates (ASRs) of breast cancer (BC) incidence in different geographic regions.

Regions with Highest BC Incidence		Regions with Lowest BC Incidence	
Australia and New Zealand	94.2	South Central Asia	25.9
Western Europe	92.6	Middle-Eastern-Western Africa	27.9
Northern Europe	90.1	South-Eastern Asia	38.1
North America	84.8	Central America	38.3

**Table 2:** Age-standardized rates (ASRs) of breast cancer (BC)-related mortality in different geographic regions.

Regions with Highest BC Mortality		Regions with Lowest BC Mortality	
Melanesia	25.5	Eastern Asia	8.6
Polynesia	21.6	Central America	10.1
Northern Africa	18.4	Australia New Zealand	12.6
Caribbean Area	18.1	North America	12.6
Western Africa	17.8	Southern America	13.4
